# Comparative effectiveness of cognitive and dynamic therapies for major depressive disorder in a community mental health setting: study protocol for a randomized non-inferiority trial

**DOI:** 10.1186/s40359-014-0047-y

**Published:** 2014-11-11

**Authors:** Mary Beth Connolly Gibbons, Rachel Mack, Jacqueline Lee, Robert Gallop, Donald Thompson, Debra Burock, Paul Crits-Christoph

**Affiliations:** Perelman School of Medicine, University of Pennsylvania, 3535 Market St (Room 649), Philadelphia, PA 19104 USA; NHS Human Services, Erdenheim, Pennsylvania USA

**Keywords:** Dynamic therapy, Cognitive therapy, Non-inferiority trial, Community mental health

## Abstract

**Background:**

There is substantial evidence that cognitive therapy is an effective intervention for the treatment of major depressive disorder. Although dynamic psychotherapies have been widely studied and are commonly practiced worldwide, there are few randomized comparisons of cognitive therapy and dynamic therapy for major depressive disorder.

**Methods:**

We are completing data collection on a randomized non-inferiority trial comparing the effectiveness of cognitive therapy and short-term dynamic psychotherapy in the treatment of major depressive disorder in the community mental health setting. Therapists employed in the community setting have been recruited for training in either short-term dynamic psychotherapy or cognitive therapy. Patients seeking services at the community site who meet criteria for major depressive disorder based on a blind independent diagnostic interview are randomized to 16 sessions of treatment. All patients are assessed at baseline and months 1, 2, 4, and 5 utilizing a comprehensive battery.

**Discussion:**

This study adds to the growing literature evaluating the effectiveness of short-term dynamic psychotherapy for specific diagnostic groups. These results will have implications for the dissemination of effective interventions for major depressive disorder in community mental health settings.

**Trial registration:**

This trial is registered at ClinicalTrials.gov, a service of the United States National Institute of Health. NIH Identifier: NCT01207271. Registered 21 September 2010.

## Background

Major depressive disorder (MDD) is a severe and disabling disorder. Recent estimates of the worldwide prevalence (current and past month) of MDD is 4.4% (Ferrari et al. 2013). In the United States, approximately 17% of individuals have an MDD episode in their lifetime (Kessler et al. 2005). Data from the Global Burden of Disease study indicates that in the U.S. in 2010, MDD was the fifth ranked disease/injury in terms of disability adjusted life years (U.S. Burden of Disease Collaborators 2013). The pain and suffering of individuals with depression and those close to them result in a heavy economic toll to this country in terms of both treatment costs and lost productivity (Greenberg et al. 2003).

There is substantial evidence supporting the efficacy of cognitive therapy (CT; Beck et al. 1979) in the treatment of MDD. The effects of CT have been shown to be equal to well-conducted pharmacotherapy (Hollon et al. 1992; Murphy et al. 1984; Rush et al. 1977; DeRubeis et al. 2005). In addition, there is evidence that CT has a relapse prevention effect (Evans et al. 1992; Kovacs et al. 1981; Simons et al. 1986; Hollon et al. 1991; Hollon et al. 2005). In fact, Hollon et al. (2005) found that CT had a relapse prevention effect that lasted beyond the end of treatment and was as effective as keeping patients on continued antidepressant medication. Finally, benchmarking and effectiveness studies have demonstrated that CT is also effective in real-world practice settings (Persons et al. 1999; Merrill et al. 2003).

Dynamically oriented psychotherapies have been and remain widely practiced in the United States and continental Europe. An international study of over 4000 psychotherapists revealed that 35.8% reported their theoretical orientation as psychodynamic; only an “eclectic” orientation was more common (Heinonen & Orlinsky 2013). Similarly, among psychologists in the U.S., a psychodynamic approach continues to be the most prevalent single theoretical orientation (Norcross & Rogan 2013).

Despite the large numbers of practicing psychologists and psychiatrists using dynamic therapy worldwide, there are few high quality randomized effectiveness studies using adequate control conditions, standardized treatments, and well-specified patient populations. Although a number of studies of dynamic psychotherapy have been conducted and reviewed (Leichsenring et al. 2014; Abbass et al. 2014; Leichsenring & Klein 2014; Gerber et al. 2011; Driessen et al. 2010; Abbass & Driessen 2010; Shedler 2010; Connolly Gibbons et al. 2008; Fonagy et al. 2005; Leichsenring 2001), very few investigations to date have met the stringent criteria laid out by Chambless and Hollon (1998) that have become the gold standard for defining psychotherapies as evidence-based. Thus, dynamic therapy for MDD has been characterized as having only “modest” research support, while CT for MDD has “strong” research support (Society for Clinical Psychology, American Psychological Association, Division 12, 2014).

Despite the lack of comparative effectiveness trials comparing dynamic therapy to CT, there persists a sense that dynamic psychotherapy is inferior to CT. In fact, a recent meta-analysis (Marcus et al. 2014) of 51 psychotherapeutic investigations concludes that there is consistent evidence supporting the superiority of CT over dynamically oriented treatments. However, these conclusions are drawn from only 3 studies that directly compare dynamic therapy to CT. Giesen-Bloo et al. (2006) found a moderate effect at 3 years in favor of schema focused therapy over transference focused psychotherapy in the treatment of borderline personality disorder. Barkham et al. (1999) found no difference between 3 sessions of either CT and dynamic therapy in the treatment of subsyndromal depression. Finally, Watzke et al. (2012) report an advantage for inpatient CT over inpatient dynamic therapy using nonmanualized interventions across common mental disorders. Despite the broad conclusions of the meta-analytic review, these three investigations hardly warrant the conclusion that there is consistent evidence of the superiority of CT and provide no evidence to support the superiority of CT for MDD specifically.

In regard to MDD, evidence meeting the strict criteria outlined by Chambless and Hollon (1998) supporting the effectiveness of short-term dynamic psychotherapy is beginning to accumulate. Such evidence is critical to daily clinical decision making and contemporary public health policy, given that this treatment is so widely practiced. Two well-designed investigations indicate that short-term dynamic psychotherapy should be considered efficacious for MDD in the context of concurrent psychotropic medication usage. Both de Jonghe et al. (2001) and Burnand et al. (2002) compared combined dynamic psychotherapy plus antidepressant treatment to antidepressant treatment alone. Both of these investigations implemented manualized dynamic psychotherapies (16 sessions in the case of de Jonghe et al. and 10 weeks in the case of Burnand et al.), randomized patients to treatments, and otherwise used sound experimental methods. Both investigations showed that the combined short-term dynamic psychotherapy and medication interventions were statistically superior to medication alone in the treatment of MDD. These investigations do not provide us with information regarding the efficacy of short-term dynamic pychotherapy when implemented alone, but they do suggest that short-term dynamic psychotherapy for MDD is an effective treatment in the context of medication, a context that is quite common in current mental health treatment. Further, the Burnand et al. (2002) investigation implemented a supportive session with a nurse in the medication alone condition, indicating that the effects may be due to the specific interventions of the dynamic treatment rather than the non-specific relationship effects of a purely supportive intervention.

More recently, Driessen et al. (2013) have presented the best evidence to date indicating that short-term dynamic psychotherapy should be considered an evidence-based psychotherapy for MDD. The Driessen et al. (2013) study, conducted in outpatient clinics in the Netherlands, was a non-inferiority trial comparing CBT to psychodynamic psychotherapy for 341 patients suffering from MDD, making it the largest randomized trial of dynamic psychotherapy for MDD published to date. The results indicate that psychodynamic psychotherapy was not inferior to CT in the treatment of MDD on measures of depression severity. Although many have drawn the conclusion that interventions are equivalent when underpowered superiority tests have failed to demonstrate statistically significant differences, this study specifically demonstrated that dynamic psychotherapy was statistically significantly non-inferior to CT. Our protocol builds upon this accumulating evidence. We have conducted a randomized comparative effectiveness trial comparing CT to short-term dynamic psychotherapy in the treatment of MDD. Like Driessen et al. (2013), this trial was designed and powered to be a non-inferiority trial. Our protocol builds on the Driessen et al. (2013) study by extending outcome assessment beyond the measurement of depression severity to include assessment of changes in functioning and quality of life and by including blind independent ratings of treatment fidelity to validate that the treatments were implemented in accordance with the treatment manuals and to demonstrate that the treatments can be distinguished.

An important distinguishing feature of our trial is that it was conducted in a community mental health setting in the U.S. In 2008, over 17 million people utilized community mental health centers (CMHCs). Thirty-five percent of these individuals were insured through Medicaid and 38% were uninsured (Wells et al. 2010). Thus, CMHCs provide service to a large number of low-income individuals within the U.S. Compared to results from efficacy trials, the treatment of MDD within the CMHC setting is less successful. Response rates for pharmacological treatment of MDD in the public sector have been reported to be less than 30% (Rush et al. 2004). Effective ways of improving the treatment of those with depressive disorders in CMHCs are therefore sorely needed. Some data on the effectiveness of CT for MDD in a CMHC setting has emerged (Merrill et al. 2003), as well as a pilot study of psychodynamic therapy (Connolly Gibbons et al. 2012), but no studies comparing different psychotherapies in the CMHC setting have been conducted to date. The generalizability of the Driessen et al. (2013) study to a low-income U.S. CMHC population, which includes a substantial ethnic minority population, is uncertain. Efforts are currently underway to disseminate CT to CMHC settings in the U.S., and such training is costly (Creed et al. 2014). Thus, it is timely to investigate whether a commonly practiced treatment (psychodynamic therapy) is inferior to CT in the treatment of MDD in the CMHC setting.

By conducting our study in a CMHC setting, unlike many efficacy trials, this trial uses patients seeking treatment and therapists providing services in the community setting. As such, the study attempts to balance internal and external validity. Although our clinicians were real world clinicians working at the community mental health site, we implemented an intensive yet flexible training program at the site to standardize the treatment delivery and ensure treatment fidelity. In addition, we included patients seeking treatment at the center and implemented few exclusion criteria to ensure that the final sample represented those receiving services in the community. However, we implemented independent blind expert ratings of diagnosis and depressive symptoms to ensure that the patient sample was well-defined and that depressive symptomatology was reliably and validly assessed.

The primary hypothesis for this trial was that short-term dynamic psychotherapy would not be inferior to CT in change in depressive symptoms for patients with MDD receiving services in a community mental health setting. Our secondary hypothesis was that short-term dynamic psychotherapy would not be inferior to CT on secondary measures of patient-rated symptoms, functioning, and quality of life. We will also investigate the mechanisms of both CT and short-term dynamic therapy in the context of the randomized trial.

## Methods

### Design

This study is a longitudinal, randomized, non-inferiority trial being conducted in a large community mental health clinic. The objective of a non-inferiority trial is to show that the new or non-established treatment is not worse than the well-established treatment by more than a pre-specified *non-inferiority margin.* This non-inferiority margin was defined for the current trial as the difference between the treatment groups that would be considered the minimum clinically relevant difference. Though most comparative studies of psychotherapies historically have used samples of 30–50 participants per treatment group, non-inferiority studies typically require larger sample sizes to conclude that one treatment is not inferior to another within the pre-specified minimal clinically meaningful difference. The larger sample size of 237 randomized patients used in the current non-inferiority trial confers an advantage over previous superiority trials: in addition to testing for non-inferiority, a separate test is also possible for superiority, and the study was designed with adequate statistical power for both tests.

### Setting: NHS human services

This trial is being conducted through a partnership with NHS Human Services (NHS), a large, non-profit, community-based organization that provides services in seven states with a concentration in the mid-Atlantic regions. NHS provides a full continuum of behavioral health services that are clinic and community based for individuals with mental health and substance abuse disorders and predominantly treats consumers receiving public assistance for these services. The current study is being conducted at an outpatient mental health clinic located just outside of Philadelphia that services approximately 4,900 patients per year and employs 80 outpatient clinicians and 3 to 4 full-time psychiatrists. This clinic serves a racially diverse population with approximately 30% of those served being African American.

### Participants

#### Patients

Patient participants for the current investigation are being recruited through the intake department at the outpatient clinic. A recruitment procedure was developed to easily identify potential participants utilizing the clinic’s existing intake procedures while not placing additional burden on the NHS intake staff who are unable to conduct extended diagnostic interviews during intake evaluations due to logistic constraints. Scores from a brief depression symptom measure, the Quick Inventory for Depressive Symptomatology (QIDS; Rush et al. 2003), are used to identify potential study participants. The cutoff score for being evaluated for the study protocol is 11. The literature indicates that QIDS scores of 11 or higher are sensitive to a score of 14 and above on the 17-item Hamilton Rating Scale for Depression (HAM-D; Hamilton 1960).

All adult patients who present at the clinic for an intake appointment complete the QIDS in the waiting room before their NHS intake assessment. The intake clinician calculates the total score and asks the patient if they would be interested in learning more about the research protocol. The intake clinician completes a short eligibility checklist for patients who scored 11 or higher on the QIDS, and attaches the checklist to the QIDS form, which is then collected by a member of the research staff. Eligibility criteria for being evaluated for the research protocol include 1) a score of 11 or above on the QIDS, 2) being between 18 and 65 years of age, 3) ability to read English at the fourth grade level and provide informed consent, and 4) willingness to be contacted for more information about study participation. Criteria that exclude patients from the study screening are 1) current or past diagnosis of schizophrenia, seizure disorder, psychotic features, and/or clinically significant organic pathology, 2) significant suicidal risk/ideation requiring immediate referral for more intensive treatment or specific gesture in the last 3 months, 3) substance/alcohol abuse symptoms requiring immediate referral to an intensive substance abuse program, and 4) pathology requiring referral to a partial hospitalization program.

After obtaining the forms from the intake department, a member of the research team reaches out to each referred patient via a telephone call. At this time, the research staff explains the purpose of the study, asks some brief screening questions, and schedules an in-person baseline assessment with interested patients who meet criteria. All baseline assessments are conducted at the NHS outpatient clinic. At the assessment, a Bachelor’s-level research assistant completes informed consent with the patient and administers the battery of self-report measures. A blind research diagnostician conducts the Structured Clinical Interview for the DSM-IV Axis I disorders (SCID) interview (First et al. 1997) to determine eligibility for study participation, and a HAM-D interview (Hamilton 1960). Patients who meet the following criteria at the baseline assessment, regardless of other diagnoses, are excluded from the study: 1) diagnosis of bipolar disorder, 2) current or past diagnosis of schizophrenia, seizure disorder, psychosis, or MDD with psychotic features, 3) depression due to organic pathology, 4) substance/alcohol abuse symptoms requiring immediate referral to an intensive substance abuse program, 5) pathology requiring immediate referral to a partial hospitalization program, and 6) score of 4 on item #3 of the HAM-D, indicating a suicidal gesture had occurred in the past week. Patients who do not meet criteria for the study are immediately referred back to the intake staff at the site for referral to a non-study therapist. After completing an informed consent, any patient who receives a current diagnosis of MDD based on the SCID interview is enrolled either as a study training case or randomized to a treatment condition. Patients in the randomization phase of the study are randomly assigned to receive up to 16 sessions across 5 months of weekly individual therapy with a clinician trained in either short-term dynamic psychotherapy or CT.

All patients who participate in the protocol receive $50 gift cards for completion of each of the baseline, month 1, month 2, and month 5 assessments and a $25 gift card for the briefer month 4 assessment.

#### Clinicians

Clinicians at the Master’s-level and above employed by NHS are recruited to be trained and treat patients in either short-term dynamic psychotherapy or CT for this study. The research staff advertise the study protocol by making announcements at staff meetings and placing fliers in the outpatient clinicians’ mailboxes. Interested clinicians meet with a member of the research staff and are enrolled in the training phase of the study if they are currently seeing adults for outpatient treatment and they anticipate remaining employed at NHS for the duration of the project. As an effectiveness trial, all therapists employed by the center are eligible to enroll in the study. Therapists are paired with treatment group based on an evaluation of their past academic training and supervision, the theoretical identity of the therapist, and stated desire to be trained in a particular treatment. Since the majority of therapists have a theoretical orientation consistent with their previous training experiences and their desired training condition, we decided to match all therapists to training condition based on their predominant orientation in order to balance treatment groups on therapist allegiance to the treatment.

The 40 therapists who have participated in training as part of this protocol to date have a variety of training backgrounds. To be employed at the center, they were required to have a Master’s degree. Degrees achieved by the therapists included in the current protocol include: Master of Arts (M.A.) in Clinical Psychology (n = 1), M.A. in Counseling Psychology (n = 3), M.A. in Clinical and Counseling Psychology (n = 2), M.A. in Community and Clinical Counseling (n = 3), M.A. in Creative Arts in Therapy (n = 1), M.A. in Health Education (n = 1), Master of Science (M.S.) in Psychology (n = 1), M.S. in Clinical Psychology (n = 1), M.S. in Counseling Psychology (n = 1), M.S. in Clinical and Counseling Psychology (n = 1), M.S. in Counseling and Clinical Health Psychology (n = 10), M.S. in Community Counseling (n = 1), M.S. in Experimental Psychology (n = 1), Master of Social Work (M.S.W.) (n = 6), Master of Social Service (M.S.S.) (n = 2), Master of Education (M.Ed.) (n = 1), and Master of Philosophy in Education (M.Phil.Ed) (n = 1). Three therapists hold doctorate degrees: Doctor of Philosophy (Ph.D.) in Educational Psychology (n = 1), Ph.D. in Social Work (n = 1), and Doctor of Education (Ed.D.) (n = 1).

### Procedures

**Figure 1:**
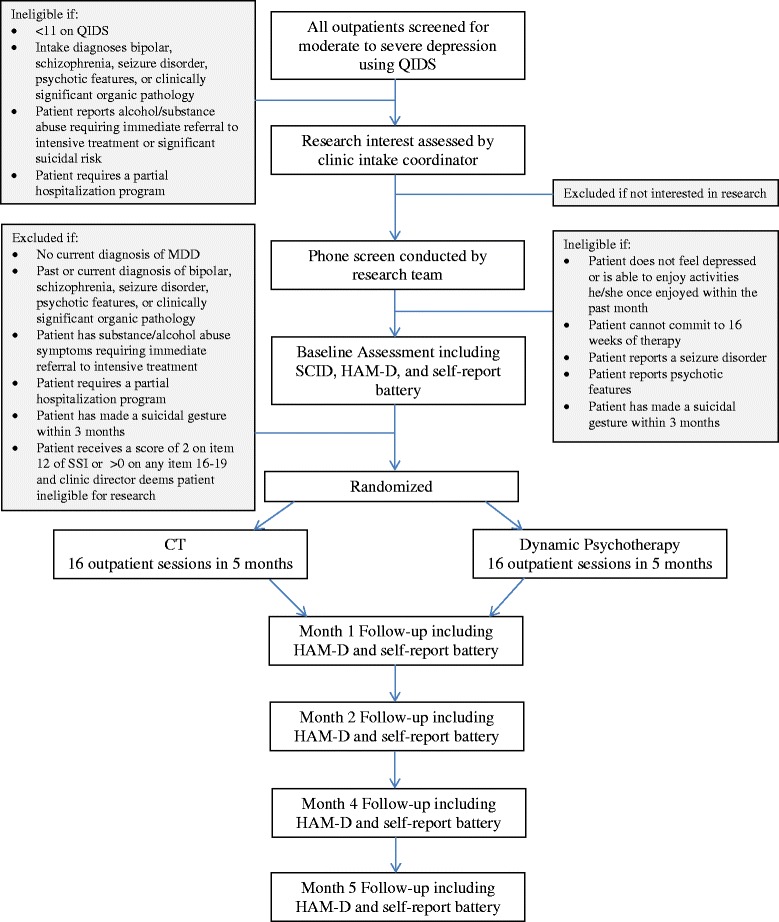
**Illustration of study design and participant flow.**

Patients seeking outpatient services at a community mental health agency are screened for moderate to severe depressive symptoms via self-report at the initial clinic intake conducted at the center. Figure 1 provides an outline of patient progress through the study protocol. Any patients with moderate to severe depressive symptoms who are interested in hearing more about the research program are referred to the research team. A member of the research team performs a telephone screen to evaluate the patient’s interest and eligibility for the study. All interested patients are scheduled for a research baseline assessment conducted by the research team at the community mental health center at the patient’s convenience. Eligible patients are randomized to either short-term dynamic psychotherapy or CT consisting of 16 weekly outpatient sessions to be completed within 5 months. All sessions take place at the CMHC and are conducted by therapists employed at the center but trained by expert research supervisors. Patients complete monthly assessments conducted by the research team at the community mental health center and meet with a member of the research team prior to each session to complete some brief self-report inventories.

### Randomization

Patient randomization is performed using a computer generated randomization algorithm in SAS to assign patients to the two treatment conditions on a 1:1 basis balancing across 7 factors. Treatments groups are balanced on gender, race (minority versus white), expectations of treatment improvement (improvement expected defined as a rating of greater than or equal to 1 on item 5 of the Attitudes and Expectations Questionnaire), whether the patient is taking psychotropic medication at treatment intake, depression severity (high severity defined as a score of greater than or equal to 20 on the 17-item HAM-D versus low defined as a score less than 20), depression recurrence (recurrence defined as 3 or more episodes of depression including the current episode based on the SCID interview versus only 1 or 2 episodes), and relationship status (currently in a long-term relationship versus not in a relationship).

### Interventions

#### Supportive-expressive dynamic psychotherapy

Supportive-expressive dynamic psychotherapy (Luborsky 1984) is a short-term dynamic psychotherapy that focuses on changes in self-understanding of interpersonal patterns as one of the main curative factors of psychotherapy. Therapists are provided with both the original manual for supportive-expressive psychotherapy (Luborsky 1984) as well as the supplemental manual elaborating clinical cases (Book 1998). The treatment begins with a number of techniques designed to build the collaborative relationship, including use of collaborative language (e.g., “we”, “us”), socialization to treatment including an exploration of the role of interpersonal patterns in the development of depressive symptoms, setting specific goals to explore a particular interpersonal pattern that is currently causing problems in the patient’s life, and repeated alliance-building techniques to ensure that the patient and therapist are working together towards a common goal. The therapist then uses interpretations and clarifications to help patients elaborate their interpersonal interactions. The therapist first helps the patient unpack current problematic interpersonal interactions that contribute to depression using the Core Conflictual Relationship Theme method (CCRT; Luborsky & Crits-Christoph 1998). The therapist helps the patient to recognize their wishes and needs towards the other person, their stereotypic ways of perceiving the response of the other person towards the patient, and their own stereotypic ways of responding in turn within each of the interpersonal interactions. The therapist then helps the patient to understand the wish and response patterns that are repetitive across relationships. Once maladaptive interpersonal patterns are identified, the therapist helps the patient to explore the history of the interpersonal patterns, to understand their own contribution to their interpersonal patterns, to explore the maladaptive nature of their patterns, and to consider alternative ways of responding to others to help fulfill their interpersonal wishes and needs.

#### Cognitive therapy

The CT is based on the standard text *Cognitive Therapy for Depression* (Beck et al. 1979) supplemented with C*ognitive Therapy: Basics and Beyond* (Beck 1995). CT consists of a series of structured sessions which target behavioral activation and disconfirmation of specific negative expectations. Standard interventions focus on activity scheduling, increasing pleasure/mastery experiences, identifying and evaluating automatic thoughts, completing dysfunctional thought records, and behavioral experiments. As treatment progresses, the emphasis shifts to the identification and evaluation of more abstract underlying beliefs and attitudes.

### Supervision and training

The design of the supervision and training protocols was driven by our desire to conduct a real world effectiveness trial while still maintaining internal validity of the treatment implementation. Especially for a non-inferiority trial, it was important that both treatments be implemented with fidelity to the treatment manuals allowing discrimination based on theoretical differences between the approaches in order for us to make conclusions about the non-inferiority of interventions. To balance the protocol design on the efficacy-effectiveness dimension, we utilize clinicians already employed at the community mental health site but use expert supervisors from the university setting with substantial clinical and research experience implementing and supervising the treatments. The short-term dynamic psychotherapy supervisor has been a clinical supervisor at the Center for Psychotherapy Research at the University of Pennsylvania for the past 20 years as well as a private practitioner with over 20 years of clinical experience. She has served as a protocol therapist and as a clinical supervisor for multiple other federally funded psychotherapy trials of dynamically oriented psychotherapy. The supervisor for the CT condition likewise has substantial clinical and research experience with CT. She has been practicing CT for 14 years, been responsible for the training of graduate students in CT, and has participated as a protocol therapist, and a co-supervisor, in multiple federally funded investigations of CT.

The training program was developed to be flexible to the needs of the community clinicians while still including the intensity of supervision necessary to achieve competent delivery of the two psychotherapeutic treatments. Following a thorough informed consent process, all clinicians are asked to read the training manuals and then attend an initial eight hour in-person training workshop with their research supervisor, a doctoral-level expert clinician in either supportive-expressive dynamic psychotherapy or CT. Workshops are delivered in 1 full day format, split across 2 sessions, or individualized into multiple sessions to meet the needs of each training cohort. The workshops are conducted at the clinical site or at the university research setting based on the preferences of the training therapists in each cohort.

After this initial workshop training, we include both intensive individual supervision across the first three cases as well as bimonthly group supervisions throughout the study to help therapists maintain what they have learned and continue to increase their therapeutic skills within the two treatment modalities. Clinicians receive one hour of individual supervision with their study clinical supervisor for every two sessions of psychotherapy delivered across each of their first three study patients. A clinician becomes eligible to treat patients in the randomized phase of the study after they complete at least eight sessions of treatment with two different training cases. The majority of the individual supervision is conducted via the telephone, with in-person meetings arranged occasionally based on the request of the supervisor and clinician. Throughout the duration of the study, all clinicians participate in one hour of group supervision with their research supervisor and fellow clinicians in their condition twice a month. The group supervisions have been conducted in person at the community mental health site across the first two months of training for each cohort until the supervisor decides they are ready to meet via teleconference.

All psychotherapy sessions are digitally recorded. Supervisors listen to at least every other psychotherapy session for each of the first three cases for each therapist to prepare for the intensive individual supervision. Additionally, supervisors listen to selected sessions from the randomized cases throughout the study to prepare for group supervision sessions. All individual and group supervision sessions focus on the review of audiotaped sessions. This protocol was also designed to be flexible to the needs of community clinicians with a variety of training backgrounds and experiences. The supervisor and clinician can request additional cases of intensive individual supervision to help a therapist achieve competence. Additional intensive individual supervision has been requested and conducted by the supervisors for two therapists in training in short-term dynamic psychotherapy and for two therapists in training in CT to date. Clinicians are reimbursed $300 for the initial training workshop and $25 for each hour of both individual and group supervision. For every two study patients who attend at least one treatment session with a given clinician, the clinician is awarded a $150 honorarium.

Unlike traditional efficacy studies, the therapist training has been conducted in waves throughout the 5 years of the study protocol in parallel to the randomization phase. In the community mental health setting, therapist turnover is high and many therapists do not remain employed at the same site for more than a few years. For this reason we trained an initial cohort of therapists during the first year, and then train yearly cohorts in each treatment condition in preparation for therapist attrition. Each new training cohort attends separate workshops and group supervision sessions until the intensive individual supervision phase is completed. At that point therapists can be rolled into ongoing group supervision groups based on the discretion of the supervisor.

As expected, the therapist turnover at the site to date has been high with the majority of therapists discontinuing the study because they were ending employment at the site. Across the 5 years of study enrollment, 22 cognitive therapists and 20 dynamic therapists have been invited to participate in a training workshop. The 22 cognitive therapists completed the workshop; 18 of the 20 dynamic therapists attended the workshop. Following the workshop, 18 cognitive therapists and 17 dynamic therapists completed intensive supervision with at least 1 training case. Of these, 9 CT therapists and 11 dynamic therapists completed the intensive supervision and have seen at least 1 patient as part of the randomized protocol. The 6 dynamic therapists who left the study during the training left for a variety of reasons, including: 3 that did not have time for the intensive yet flexible supervision schedule, 1 did not want to continue the supervision, and 2 that were satisfied with the training and study but left employment at NHS. Similarly, 3 CT therapists discontinued during training because they were too busy, 3 did not feel comfortable with the supervision, 1 felt that CT was not a good match, 1 was discontinued by NHS, and 1 left employment at the site.

### Fidelity

We assess adherence and competence of both the CT and short-term dynamic interventions in order to evaluate the fidelity with which the interventions are administered and to confirm that treatments can be discriminated as practiced in the community mental health setting. Four advanced graduate students with training in CT have been recruited to rate adherence and competence of CT. Four additional advanced graduate students with training in dynamic psychotherapy are employed to rate adherence and competence to short-term dynamic psychotherapy. All judges have been trained to reliability across 4 training cases and continue with monthly recalibration sessions to maintain inter-rater reliability. One session from each patient from the early sessions of treatment (usually session 3) is rated by 3 judges using a balanced incomplete block design. The CT judges rate sessions from all CT cases and a random selection of 20 cases from the dynamic condition. Alternatively, the trained dynamic judges rate a session from all short-term dynamic cases and a random selection of 20 cases from the CT condition. All judges complete ratings independently and are blind to the study design.

For adherence and competence to short-term dynamic therapy, we are using an adaptation of the Adherence/Competence Rating Scale for Supportive Expressive Dynamic Psychotherapy (Barber & Crits-Christoph 1996) specifically adapted to the community friendly dynamic intervention utilized in the current investigation (Connolly Gibbons et al. 2012). Items representing the techniques included in the supportive expressive model are rated on a 7-point scale for adherence defined as how frequently an intervention was used, and are rated on a 7-point competence scale representing how well the intervention is implemented.

For fidelity ratings of CT, the trained independent judges are using both the CT subscale of the Collaborative Study Psychotherapy Rating Scale (Hill et al. 1992) to rate therapist adherence to CT techniques and the Cognitive Therapy Scale (Vallis et al. 1986) to measure competence in delivering CT interventions.

### Measures

#### Overview

All participants complete a baseline diagnostic and HAM-D interview conducted by a trained advanced graduate student research diagnostician blind to the study design as well as a comprehensive battery of self-report measures covering demographic characteristics, treatment expectations, depressive symptoms, functioning, quality of life, interpersonal problems, trauma history, and alcohol and substance use. All participants also complete monthly assessments at months 1, 2, 4, and 5 consisting of a HAM-D interview and the self-report battery. At monthly assessments, the research diagnosticians are blind to treatment condition and number of sessions of treatment attended. In addition, patients complete measures of mechanism of change at the months 1, 2, and 5, including measures of dysfunctional attitudes, compensatory skills, depressotypic schemas, and self-understanding of interpersonal patterns. Patients complete the 24-Item Behavior and Symptom Identification Scale (BASIS-24; Eisen et al. 2004) and the Beck Depression Inventory-II (BDI-II; Beck et al. 1996) prior to each session, the measure of alliance following sessions 2, 4, 6, 8, and the measure of treatment credibility after session 2. A summary of the assessment battery is provided in Table 1.

**Table 1:** **Summary of study measures**

**Study variables**	**Assessment point**	**Measurement scale**	**Reference**
*Diagnosis*			
Depression	Baseline	Quick Inventory for Depressive Symptomatology (QIDS), 16 items, α = .86	Rush et al. 2003
Depression	Baseline	Structured Clinical Interview (SCID) for the DSM-IV	First et al. 1997
*Clinical characteristics*			
Demographics	Baseline	Demographic Questionnaire	Developed for current study
Alcohol/substance use	Baseline	Alcohol Use Disorders Identification Test (AUDIT), 10 items, α = .83	Hays et al. 1995
		We developed 6 items to evaluate substance use, adapted from the AUDIT	
Trauma history	Baseline	Traumatic Life Events Questionnaire (TLEQ), 24 items	Kubany et al. 2000
Treatment expectations	Baseline	Attitudes and Expectations (AAE), 14 items	Adapted from Elkin et al. 1989
*Symptoms*			
General mental health	Baseline, M1, M2, M4, M5, T1-16	Behavior and Symptom Identification Scale (BASIS-24), 24 items, α = .77-.91	Eisen et al. 2004
Depression	Baseline, M1, M2, M4, M5, T1-16	Beck Depression Inventory–II (BDI-II), 21 items, α = .91	Beck et al. 1996
Depression	Baseline, M1, M2, M4, M5	Hamilton Rating Scale for Depression (HAM-D), 27 items, α = .79	Hamilton, 1960; Trajkovic et al. 2011
*Functioning/broader outcome*			
Life satisfaction	Baseline, M1, M2, M4, M5	Quality of Life Inventory (QOLI), 32 items, α = .77-.89	Frisch et al. 1992
General functioning	Baseline, M1, M2, M4, M5	The Medical Outcomes Study 36-item Short-Form (SF-36), 36 items, α = .78-.94	Ware & Sherbourne 1992; McHorney et al. 1994
Interpersonal problems	Baseline, M1, M2, M4, M5	Inventory of Interpersonal Problems (IIP-48), 48 items, α = .69-.80	Gude et al. 2000
*Therapeutic process*			
Therapeutic alliance	T2, T4, T6, T8	Working Alliance Inventory–Client (WAI-C), 12 items, α = .93	Horvath & Greenberg 1989
Treatment credibility	T2	Opinions About Treatment (OAT), 3 items, α = .90	Adapted from Borkovec & Nau 1972; Mooney et al. 2013
*Therapeutic mechanism*			
Dysfunctional attitudes	Baseline, M1, M2, M5	Dysfunctional Attitudes Scale (DAS), 40 items, α = .86	Weissman & Beck 1978; De Graaf et al. 2009
Compensatory skills	Baseline, M1, M2, M5	Ways of Responding–Community (WOR-C), α = .72-.79	Adapted from Barber & DeRubeis 1992; Connolly Gibbons et al. 2014
		Ways of Responding–Self-Report (WOR-SR), 65 items, α = .94-.96	
Self-understanding	Baseline, M1, M2, M5	Self-Understanding of Interpersonal Patterns Scale–Revised (SUIP-R), 28 items, α = .92	Connolly Gibbons et al. 2009
Underlying depressogenic schemas	Baseline, M1, M2, M5	Psychological Distance Scaling Task (PDST), 80 items	Dozois & Dobson 2001

*Note*: M indicates month assessment (M1 = month 1). T indicates therapy session (T1 = therapy session 1).

#### Quick Inventory of Depressive Symptomatology-Self-Report (QIDS)

The QIDS (Rush et al. 2003) is a 16-item, self-report measure designed to assess the severity of depressive symptoms using the criterion symptoms designated by the DSM-IV. The QIDS has demonstrated good internal consistency (Cronbach’s α = .86) in patients with chronic major depression (Rush et al. 2003). In this same sample, total scores on the QIDS were highly correlated (*r =* .81*)* with the 17-item HAM-D (Rush et al. 2003).

#### The Structured Clinical Interview for DSM-IV (SCID)

The SCID-I (First et al. 1997) for DSM-IV was used to diagnose MDD. For MDD, the kappa coefficient ranges from .61-.93 (Lobbestael et al. 2011; Segal et al. 1995; Skre et al. 1991; Williams et al. 1992; Zanarini & Frankenburg, 2001; Zanarini et al. 2000).

#### The Hamilton Rating Scale for Depression (HAM-D)

The HAM-D (Hamilton 1960) is a widely used inventory for evaluating the severity of common symptoms of depression. The 24-item version of the HAM-D was completed by applying the Structured Interview Guide to enhance reliability (Williams 1988). A recent meta-analysis reports a Cronbach’s alpha of .79, as well as good inter-rater and test-retest reliability (Trajkovic et al. 2011).

#### The 24-Item Behavior and Symptom Identification Scale (BASIS-24)

The BASIS-24 (Eisen et al. 2004) is a 24-item self-report inventory designed to measure mental health status from the consumer’s point of view. The items cover six domains including: depression/functioning, interpersonal relationships, psychotic symptoms, alcohol/drug use, and emotional lability. The measure has demonstrated acceptable test-retest reliability and internal consistency and good construct and discriminant validity (Eisen et al. 2004). Further studies have supported the reliability, concurrent validity, and sensitivity of the BASIS-24 in specific racial groups (Eisen et al. 2006).

#### Beck Depression Inventory-II (BDI-II)

The BDI-II (Beck et al. 1996) is a 21-item, self-report questionnaire designed to assess recent depressive symptoms. The measure demonstrates high internal consistency (α = .92) and adequate convergent and discriminant validity (Beck et al. 1996).

#### Quality of Life Inventory (QOLI)

The QOLI (Frisch et al. 1992) is a 32-item self-report measure which first rates the importance of something in a person’s life, such as money or self-esteem, and then rates how satisfied a person is with this item. The measure demonstrates good internal consistency (α = .77-.89) and good convergent validity with seven other measures of well-being and life satisfaction. The QOLI also demonstrates good test-retest reliability (*r* = .80-.91) (Frisch et al. 1992).

#### The Medical Outcomes Study 36-Item Short Form (SF-36)

The SF-36 (Ware & Sherbourne, 1992) is a widely used standardized, generic self-report of health status for evaluating physical and mental health-related quality of life. The SF-36 consists of 36 items; 35 of the items group into eight multi-item scales that collectively measure health-related quality of life (Physical Functioning, Role Limitations due to Physical Health Problems, Bodily Pain, General Health Perceptions, Vitality, Social Functioning, Role Limitations due to Emotional Problems, and Mental Health), and the remaining item concerns the experience of change in general health during the last year. The measure demonstrates good internal consistency for each of the eight scales, with Cronbach’s alpha ranging from .78 for General Health Perceptions to .93 for Physical Functioning (McHorney et al. 1994).

#### Pretreatment expectations

To assess pretreatment expectations we utilize one item from an unpublished measure (Moras & Jones 1992) adapted from the National Institute of Mental Health Treatment of Depression Collaborative Research Program (Elkin et al. 1989). The item asks participants to rate the question, “How much improvement do you expect to experience as a result of treatment,” on a 7-point Likert scale ranging from “-3” (I expect to feel much worse) to “3” (I expect to feel much better). The use of the single item measure of pretreatment expectations has been widely used in previous studies and has shown validity in predicting therapeutic alliance (Connolly Gibbons et al. 2003) and treatment credibility (Mooney et al. 2013).

#### Treatment credibility form: Opinions About Treatment (OAT)

This questionnaire is administered early in the treatment to obtain the patient's opinion of the probable value of the treatment he/she is receiving for his/her problems. It will be used to determine whether the treatment approaches are equal in credibility. The OAT (Borkovec & Nau 1972) demonstrates high internal consistency (α = .90) (Mooney et al. 2013).

#### The Inventory of Interpersonal Problems-48 (IIP-48)

This 48-item self-report measure helps to identify sources of interpersonal distress. It describes both the types of interpersonal problems that people experience and the level of distress associated with them and can be used to measure interpersonal changes over the course of therapy. The IIP-48 (Gude et al. 2000) measures Assertiveness, Sociability, and Interpersonal Sensitivity. It demonstrates good internal consistency (α = .69-.80) and high correlation with the original 127-item IIP (Horowitz et al. 1988).

#### Working Alliance Inventory–Client (WAI-C)

This 12-item self-report form measures the quality of the alliance between the therapist and the patient from the point of view of the patient. The WAI-C (Horvath & Greenberg 1989) demonstrates high internal consistency (α = .93) and good convergent validity with the Counselor Rating Form (CRF; LaCrosse & Barak 1976) and the Empathy Scale of the Relationship Inventory (RI; Barrett-Lennard 1962).

#### Traumatic Life Events Questionnaire (TLEQ)

This 24-item questionnaire assesses exposure to a broad range of potentially traumatic events and provides a brief trauma history of the patient. When events are endorsed, respondents are asked if they experienced intense fear, helplessness, or horror. The TLEQ demonstrates good temporal stability, discriminant validity, and construct validity across all subscales (Kubany et al. 2000).

#### Alcohol Use Disorders Identification Test (AUDIT)

This test was developed as a screening instrument for hazardous and harmful alcohol consumption. It is a 10-item questionnaire which covers the domains of alcohol consumption, drinking behavior, and alcohol-related problems. The research team constructed a parallel assessment of substance use also covering consumption, behavior, and substance-related problems. The AUDIT (Saunders et al. 1993) demonstrates good internal consistency (α = .83) and acceptable convergent validity with the CAGE alcohol screen (r = .62) and the Michigan Alcoholism Screening Test (MAST) (*r* = .66) (Hays et al. 1995).

#### Dysfunctional Attitudes Scale (DAS)

The DAS (Weissman & Beck 1978) is a 40-item instrument that is designed to identify and measure cognitive distortions, particularly distortions that may relate to or cause depression. The items contained on the DAS are based on Beck’s cognitive therapy model and present 7 major value systems: Approval, Love, Achievement, Perfectionism, Entitlement, Omnipotence, and Autonomy. The measure demonstrates good internal consistency (α = .86) and acceptable convergent validity. Depressed individuals score significantly higher on both Perfectionism and the 17-item total than their non-depressed counterparts. Further, the Perfectionism score and 17-item total score correlate more highly with depression severity than with dependency (De Graaf et al. 2009).

#### Ways of Responding–Community (WOR-C)

We adapted the Ways of Responding (WOR; Barber & DeRubeis 1992) for use in the community mental health setting. The WOR is a 6-item essay response questionnaire which measures compensatory skills through mood inductions. After each mood induction scenario is presented, the subject writes how he or she would respond in the given situation. The WOR-C (Connolly Gibbons et al. 2014) was adapted from the WOR by developing depressive scenarios that were more applicable to the community population. The WOR-C demonstrated good reliability and convergent validity with the original WOR and other measures of depressotypic thinking in both student normal and clinical samples (Connolly Gibbons et al. 2014). Four graduate student judges have been trained to categorize each thought unit provided by the patient into a set of possible depressotypic cognitive behavioral responses or compensatory cognitive skills across 4 training sessions held via teleconference. Three judges are assigned to blindly rate each patient scenario using a balanced incomplete block design. While ratings are being completed, monthly recalibration sessions are used to prevent rater drift.

#### Ways of Responding–Self–Report (WOR-SR)

The WOR-SR (Connolly Gibbons et al. 2014) is a 65-item measure adapted from the WOR modifying the measure to capture compensatory skills using a self-report Likert scale format. The WOR-SR demonstrated good internal consistency reliability and convergent validity in both student normal and clinical samples (Connolly Gibbons et al. 2014).

#### Psychological Distance Scaling Task (PDST)

The PDST (Dozois & Dobson 2001) is a measure of cognitive organization that captures underlying depressogenic schemas. On this task, a square grid is presented to subjects on a computer monitor. In the middle of this grid is a horizontal line, anchored with the statement *Not at all like me* on the left and *Very much like me* on the right. A vertical line is also shown in the middle of the grid with the anchors *Very positive* at the top and *Very negative* at the bottom. The subject is presented with 80 different adjectives (one at a time) in the center of the grid, and respondents are instructed to move the mouse to the position on the screen that best characterizes the degree of self-relevance and degree of valence of the word.

#### Self-Understanding of Interpersonal Patterns-Revised (SUIP-R)

The SUIP-R (Connolly Gibbons et al. 2009) is a 28-item self-report instrument that measures a client’s level of understanding of his or her interpersonal patterns. The self-understanding score represents each patient’s level of self-understanding of those interpersonal conflicts that are relevant in his/her world. Self-understanding is defined across a continuum ranging from recognition of an interpersonal conflict within a specific situation, to recognition of the pervasiveness of the pattern, the history of the pattern, one’s own contribution to the pattern, and finally to the ability to recognize and then reevaluate the pattern at the time it is experienced. The SUIP-R has demonstrated good internal consistency, convergent validity, sensitivity to change, and ability to discriminate change in dynamic psychotherapy compared to other therapeutic conditions (Connolly Gibbons et al. 2009).

### Diagnosticians

Nine advanced graduate students in clinical psychology have been hired by the research team to conduct the SCID and HAM-D interviews. All diagnosticians are blind to the treatment conditions being implemented in the protocol as well as all study hypotheses. The diagnosticians are scheduled by the research team for all assessment appointments and conduct all assessments in person at the community mental health center. Training procedures for diagnosticians are tailored to each diagnostician’s entry skill level. All diagnostic training has been conducted and supervised by a master’s-level clinician with substantial experience conducting both SCID and HAM-D interviews as well as substantial experience training diagnosticians to competence. The diagnostic training procedure has five components: (a) a training workshop with the diagnostic supervisor to review diagnostic procedures; (b) rating SCID master training tapes prepared by Miriam Gibbon, M.S.W., and Michael First, M.D., of New York State Psychiatric Institute and reviewing ratings with the diagnostic supervisor; (c) rating audiotapes of diagnostic interviews conducted by experienced diagnosticians to obtain a preliminary estimate of the reliability with which the new diagnostician interprets SCID-obtained data as evidence for DSM diagnostic criteria; (d) conducting a diagnostic interview on a mock patient and reviewing the results with the diagnostic supervisor; and (e) conducting 2 training interviews at the community site with extensive review and feedback from the diagnostic supervisor. All diagnostic training interviews are conducted on training cases for the current protocol. Diagnostic interviews for randomized cases are only conducted by diagnosticians who have completed these training steps and are deemed competent by the diagnostic supervisor. The diagnostic supervisor then conducts monthly group conference calls with all diagnosticians to review problems, questions, and concerns. These conferences are further used to review a diagnostic interview with the diagnosticians to maintain reliability. Finally, the expert diagnostician conducts a random review of 10% of the audiotapes of diagnostic interviews and provides written feedback to the diagnostician to prevent drift.

### Study progress

This study began enrollment on October 10th, 2010 and the target enrollment of 237 patients was completed on July 2nd, 2014. This study was powered to randomize 230 patients to the two treatment condition, estimating that 203 patients would have at least one post baseline assessment. To date we have randomized 237 patients to the two treatments and have 208 patients with at least one post-baseline assessment. Recruitment for this protocol has closed with 7 patients still engaged in active treatment and 16 patients still due for follow-up assessments. Data collection is expected to be completed by December, 2014.

### Statistical analysis

Preliminary data analyses will include descriptive statistics and exploratory graphing such as frequencies, means, standard deviations, box and whisker plots, stem and leaf diagrams, and scatter plots to assess the normality of the data in terms of the presence of skew and/or outliers for both the outcomes and adherence scores. If necessary, the continuous outcome data will be transformed by using an appropriate transformation such as the log transformation for skewed, long-tailed data.

#### Hypothesis 1: short-term dynamic psychotherapy will be not inferior to CT on change from baseline to endpoint of acute treatment in the HAM-D total score

This hypothesis is formulated in terms of a non-inferiority test, which is a one-sided equivalence test. The non-inferiority margin will be established to be smaller than any clinically relevant change and will be based on the recommendation for this provided by McHorney et al. (1994) and previously implemented in a non-inferiority study of treatments for MDD (Szegedi et al. 2005): a non-inferiority margin of 2.5 HAM-D total score points (this is the difference in change from baseline to endpoint between the two treatment groups). Standard deviation of change scores on the HAM-D are not typically reported in published studies, but standard deviations from two large studies of CT ranged from 6.4 (from Dimidjian et al. 2006) to 6.8 (DeRubeis et al. 2005). However, one published study of medication for MDD found a standard deviation of 8.5 (Szegedi et al. 2005). Although the studies with CT groups had lower standard deviations, these studies were not done in community settings where variability would be expected to be higher. Thus, to be conservative, we will use the higher figure (8.5) from the Szegedi et al. (2005) study. Based on the 8.5 standard deviation of HAM-D total score change scores, the 2.5 HAM-D points corresponds to a Cohen’s *d* effect size of 0.29.

We expect that some patients will fail to complete the study. We will implement pattern mixture models to assess the randomness of the “completion” process. If the process is random, we will implement the modified two one-sided test approach for equivalence discussed by Lee et al. (2005), who discussed a maximum likelihood statistical procedure for analyzing equivalence trials with missing observations.

Secondary analyses will also be conducted using the mixed effects model to test for slope differences between treatment groups in the HAM-D. The mixed effects modeling is used to account for the clustering structure of the data (i.e., repeated assessments within an individual) and is implemented with the SAS Mixed Procedure of the SAS 9.1.3 software. We will implement a mixed-model analysis of variance (MMANOVA). The MMANOVA does not assume any specific profile/pattern between the outcome variable and time, but rather focuses on the average separation between groups across the treatment period. We will use baseline assessment as a covariate, and assess the average outcome between groups across the post-baseline monthly assessments. The MMANOVA is similar to a repeated measures analysis of variance model, but offers flexibility to deal with missing data and modeling the variance-covariance matrix of the outcome (Schwarz 1993).

All modeling structures allow for important covariates. We will include any baseline measures which differ between groups. The impact of gender and minority status on any treatment effects will also be explored in secondary analyses.

#### Hypothesis 2: short-term dynamic psychotherapy will be not inferior to CT on change from baseline to endpoint of acute treatment on secondary measures of symptoms, patient functioning, and quality of life

Analyses of the secondary outcome measures will be conducted as non-inferiority analyses as described above for the primary outcome measure. For the secondary analyses, we will use the BASIS-24 total score, the Physical Component Summary and the Mental Component Summary of the SF-36, as well as the total score from the QOLI. Because there is no consensual definition of a minimally clinically significant difference on these other measures, we will set the non-inferiority margin to be the same effect size (*d* =0.29) used for the HAM-D. We will also conduct analyses using the mixed effects model to test for slope differences between treatment groups on each of the secondary outcome measures, with only the number of assessments varying depending on the measure.

### Ethics statement

All study procedures are being conducted in compliance with the Institutional Review Board of the University of Pennsylvania.

### Data Safety Monitoring Board (DSMB)

A DSMB was appointed to monitor the progress of the study, review data quality, and evaluate patient safety issues as they arise. The committee consists of three scientists who are independent of the study.

## Discussion

The results of this investigation will supplement the growing body of literature evaluating the effectiveness of short-term dynamic psychotherapy for the treatment of MDD. This trial builds on the non-inferiority trial published by Driessen et al. (2013) by extending the comparison of CT and short-term dynamic psychotherapy to the U.S. community mental health setting utilizing primarily clinicians with master’s degrees, by going beyond symptom outcomes to include both patient functioning and quality of life, and by implementing blind and independent adherence/competence ratings to ensure the treatments were delivered with fidelity. To date, there is substantial evidence indicating that CT is an efficacious intervention for the treatment of MDD. Our results should add to the literature evaluating whether short-term dynamic psychotherapy might also be considered an efficacious alternative intervention for the treatment of MDD in the CMHC setting.

This trial also has limitations that should be considered in evaluating the results. The design as a non-inferiority trial has the significant advantage of allowing us to evaluate whether the treatments are statistically equivalent in effectiveness. By comparing the effects of short-term dynamic psychotherapy to an already established treatment, we can evaluate the equivalence of these treatments in practice. However, our design did not include a control condition to control for the passage of time. In addition, our study generalizes to the community mental health setting. Although it is useful to study the comparability of these treatments in the real world of treatment delivery, it is possible that the results will not generalize to other practice settings.

In conclusion, this randomized non-inferiority trial will add to our understanding of the effectiveness of short-term dynamic psychotherapy as a treatment alternative for MDD. This research focus could have implications for the dissemination of interventions for the treatment of MDD in the real world. To date, tremendous efforts and resources have been spent retraining therapists and disseminating CT for the treatment of MDD despite the fact that many therapists practicing worldwide describe themselves as dynamically oriented. The comparability of these treatments for MDD in real world practice should be considered in dissemination efforts.

## Abbreviations

AUDIT: Alcohol use disorders identification test
BASIS-24: 24-Item behavior and symptom identification scale
BDI-II: Beck depression inventory-II
CCRT: Core conflictual relationship theme
CMHC: Community mental health center
CRF: Counselor rating form
CT: Cognitive therapy
DAS: Dysfunctional attitudes scale
HAM-D: Hamilton rating scale for depression
IIP: Inventory of interpersonal problems
MAST: Michigan alcoholism screening test
MDD: Major depressive disorder
MMANOVA: Mixed-model analysis of variance
OAT: Opinions about treatment
PDST: Psychological distance scaling task
QIDS: Quick inventory for depressive symptomatology
QOLI: Quality of life inventory
RI: Relationship inventory
SCID: Structured clinical interview for DSM-IV
SF-36: Medical outcomes study 36-item short form
SUIP-R: Self-understanding of interpersonal problems-revised
TLEQ: Traumatic life events questionnaire
WAI-C: Working alliance inventory-client
WOR-C: Ways of responding-community
WOR-SR: Ways of responding-self report
